# A potential role of alternative splicing in the regulation of the transcriptional activity of human GLI2 in gonadal tissues

**DOI:** 10.1186/1471-2199-7-13

**Published:** 2006-03-23

**Authors:** Mart Speek, Olga Njunkova, Illar Pata, Eola Valdre, Priit Kogerman

**Affiliations:** 1 Department of Gene Technology, Tallinn University of Technology, Akadeemia tee 15, 19086 Tallinn, Estonia; 2 Department of Molecular Genetics, National Institute for Chemical Physics and Biophysics, Akadeemia tee 23, 12618 Tallinn, Estonia; 3 Diagnostic Clinic, East-Tallinn Central Hospital, Ravi 18, 10138 Tallinn, Estonia

## Abstract

**Background:**

Mammalian Gli proteins are important transcription factors involved in the regulation of Sonic hedgehog signal transduction pathway. Association of Gli2 with mammalian development and human disease led us to study the structure and expression of the human *GLI2*.

**Results:**

We show that the region encoding GLI2 repressor domain is subject to alternative splicing in the gonadal tissues and different cell lines. Two major alternatively spliced forms of *GLI2 *mRNA arise from skipping exon 3 (*GLI2*Δ3) or exons 4 and 5 (*GLI2*Δ4–5). Both forms contain premature translational stop codons in the *GLI2 *open reading frame (ORF) starting from exon 2. Translation of *GLI2*Δ3 and *GLI2*Δ4–5 *in vitro*, initiated from downstream AUG codons, produced N-terminally truncated proteins. In Gli-dependent transactivation assay, expression of *GLI2*Δ3 induced activation of the reporter gene similar to that of the full-length construct (*GLI2*fl) containing complete ORF. However, expression of the *GLI2*Δ4–5 resulted in about 10-fold increase in activation, suggesting that deletion of the major part of repressor domain was responsible for the enhanced activation of GLI2 protein.

**Conclusion:**

Our data suggest that in addition to proteolytic processing, alternative splicing may be another important regulatory mechanism for the modulation of repressor and activator properties of GLI2 protein.

## Background

Segment polarity genes induce signaling pathways that direct morphogenesis by giving cells positional information that in turn is translated into appropriate differentiation programs. The Sonic hedgehog (Shh) signaling pathway is required in many tissues for embryonic patterning, cell proliferation and differentiation [1-3]. Inappropriate activation of the pathway drives tumorigenesis in the skin [4-8] and other tissues [9-11].

The Cubitus interruptus protein (Ci) in *Drosophila *and Gli proteins in mammals are the transcriptional effectors of the Shh signaling pathway. Like in fruit fly, multiple Gli transcription factors in vertebrates participate in the transduction of Shh signal and may repress transcription of Shh target genes [12,13]. Similarly to Ci, Gli2 and Gli3 can be proteolytically processed forming an N-terminal repressor that is concentrated in the nucleus [12-15]. Interestingly, deletion of N-terminal fragment of mouse Gli2 containing putative repressor domain altered skin tumor phenotype [5]. Hedgehog (Hh) signaling controls Ci protein activity at the post-translational level. In the absence of the Hh signaling Ci is processed into a truncated repressor form which can inhibit Hh target genes [16]. Loss of Hh function results in all Ci being converted into the repressor form [17]. Different *in vitro *functions of Gli proteins suggest that Gli2 and Gli3 respond to and are activated by Shh signaling, whereas Gli1 is a transcriptional target of activated Gli2 and Gli3 [12,18].

Several studies reveal how Gli proteins are regulated in the cytoplasm through vertebrate protein Supressor of fused (Sufu), previously identified in flies as having antagonistic role in Hh signaling [19-21]. Sufu can sequester Gli proteins in the cytoplasm, but can also interact with Gli bound to DNA. Thus, Sufu is considered to be a key negative regulator of the Hh signaling pathway in vertebrates [20]. Targeted disruption of the murine suppressor of fused gene (Sufu) led to a phenotype that included neural tube defects and lethality at mid-gestation [22].

It has been proposed that Hh signaling leads to the inhibition of Sufu, deposphorylation of Glis and the production of transcriptionally active forms with enhanced nuclear import [23]. A short motif of four amino acids (aa), SYGH, is required for the interaction of Sufu with Gli. The activity of Gli transcription factors with mutations in this motif is no longer suppressed by co-expression with SUFU [21].

Each Gli has distinct activities that are analogous to the regulatory properties of Ci [13]. The first studies on mammalian *Gli *genes *in vivo *revealed the combinatorial action of genes. In fact, Gli1 and Gli2, but not Gli1 and Gli3 have extensive overlapping functions [24,25]. Gli2, but not Gli1, is required for initial Shh signaling and ectopic activation of Shh pathway [26]. *Gli2*^-/- ^mice die at birth exhibiting defects in floor plate and adjacent interneuron development, as well as in vertebrae, bones and lungs [1,3,27,28]. Interestingly, *Gli2;Gli3 *double mutant mice develop more severe defects in skeleton and foregut derivatives than either single mutant, indicating that *Gli2 *and *Gli3 *possess both unique and overlapping functions [3,27]. In addition, loss-of-function mutations in the human *GLI2 *gene are associated with a distinctive phenotype whose primary features include defective anterior pituitary formation and pan-hypopituitarism, with or without overt forebrain cleavage abnormalities [29]. Similarly, several disorders of mouse and human development, are caused by *GLI3 *mutations [30,31] and references therein.

Despite of extensive gene targeting studies, there have been no comprehensive studies on the structure of the *Gli2 *gene. Human GLI2 was originally identified as a Tax-helper protein (THP) that binds to Tax-responsive element in the long terminal repeat of the human T-cell leukemia virus [32]. However, when compared to orthologous *Gli2 *genes from different species, the human mRNAs lacked a part of the 5' region encoding the evolutionarily conserved N-terminus of Gli2. Recently, Roessler et al. [33] have discovered a 5' sequence encoding 328 aa and showed that this so far undescribed amino-terminal repressor domain was essential for the dominant negative activity of the human GLI2. The transcription repression activity of C-terminally truncated Gli variants has been demonstrated by two independent studies showing that Gli2 and Gli3 proteins contained separate transcription repressor and activator domains [12] which in case of Gli3 were regulated by proteolytic processing [12,14,15]. However, in contrast to Gli3, overexpressed Gli2 was not processed efficiently [13]. Thus, the exact mechanism of repressor generation remains unclear, leaving us with a question whether mechanisms other than proteolytic processing may influence the functional activity of Gli2.

To study the potential role of mRNA splicing in generation of different GLI2 protein variants, we determined the exon-intron organization of human *GLI2 *and analyzed tissue-specific distribution of *GLI2 *mRNA and its alternatively spliced forms. Here we show that the revised human *GLI2 *contains an alternative 5' noncoding exon and its last coding exon encompasses a 1822 bp-long 3' UTR. Comparison with the mouse *Gli2 *gene/mRNA confirmed the presence of exons 3–6 in the human *GLI2 *[33]. Two novel alternatively spliced forms of *GLI2 *generated by skipping exon 3, or exons 4 and 5 were detected in ovary and testis. These forms showed different activator properties in the GLI-dependent transactivation assay. Our results suggest that alternative splicing in the 5' terminal region of human *GLI2 *mRNA plays an important role in regulation of GLI2 expression and generation of protein isoforms with different activities.

## Results

### Human *GLI2 *contains four exons encoding amino-terminal repressor domain

**Figure 1:**
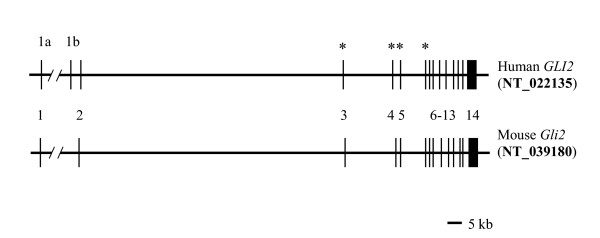
**Exon-intron organization of the human and mouse *Gli2 *genes**. Gene structures were predicted from the mapping of mouse and human *GLI2 *mRNAs to the corresponding genomic sequences (GenBank reference accession numbers shown on the right). Exons (numbered) and introns are shown by vertical and horizontal lines, respectively. Alternative 5' noncoding exons in human gene are designated as 1a and 1b. Asterisks indicate exons 3–6, predicted from the comparison of mouse and human Gli2 mRNAs and genomic structures, identified by Roessler et al. [33].

To identify common structural elements of the human and mouse *Gli2 *genes, we compared their genomic structures and mRNAs. Mouse *Gli2 *gene contains 14 exons located in about 220 kb region on the chromosome 1E2+3, as revealed by alignment of the mouse *Gli2 *mRNA [GenBank: X99104][34] to the corresponding genomic contig [GenBank: NT_039180](Fig. 1). Extension of the published mRNA sequence [34] by 180 nucleotides (nt) in the 5' untranslated region (UTR) and 543 nt in the 3' UTR using overlapping EST sequences [GenBank: CN536241 and AW546128/BC031171] generated mRNA of 6576 nt. The last exon has 1651 nt of 3' UTR and contains a polyadenylation signal ATTAAA, located 15 nt upstream of the polyA addition site. The location of the cap site of the mRNA is not known. Mouse *Gli2 *mRNA has a coding region of 1544 aa predicting a protein of 165 kD [34].

To characterize the exon-intron organization of human *GLI2*, we aligned human ESTs [GenBank: BM147847, CN295561, AI822132, BX103004 and AI089685] and human and mouse *Gli2 *mRNAs [GenBank: NM_030379 and X99104] to the human genomic contig [GenBank: NT_022135]. These alignments revealed that, similarly to the mouse counterpart, human *GLI2 *consists of 14 exons spanning 250 kb on the chromosome 2q14 (Fig. 1). Comparison between mouse and human *Gli2 *structures predicted the presence of exons 3–6 of human *GLI2 *similar to those of the mouse *Gli2*. This conclusion confirms earlier finding of Roessler et al. [33], who demonstrated the presence of exons 3–6 in the human *GLI2 *mRNA, although details of their prediction have not been described. The complete ORF of human *GLI2 *starts from exon 2 and terminates in exon 14 predicting a protein of 1569 aa with molecular weight of 166 kD (Fig. 4). The predicted protein structure differs from that described by Roessler et al. [33] by a17 aa sequence (GQVSGHGSCGCALPLSQ). This extra sequence is present in the published protein sequence [GenBank: AAY87165] that is derived from alternatively spliced *GLI2 *mRNA [GenBank: DQ086814] [33]. The respective mRNA is spliced using alternative acceptor splice site located 51 nt upstream from the boundary of intron 8 and exon 9. It should be noted that alternative splicing involving 51 nt deletion/insertion in the coding region of *GLI2 *mRNA has been described previously [32]. Since our RT-PCR reaction using primers derived from exons 2 and 14 (see Methods) yielded the major product of 2481 bp lacking the 51 nt in exon 9, we believe that the GLI2 protein used in our studies represents the predominant form (1569 aa) of the human GLI2 protein. In addition, mouse *Gli2 *mRNA coding region [34] lacks the respective 51 nt sequence.

**Figure 2:**
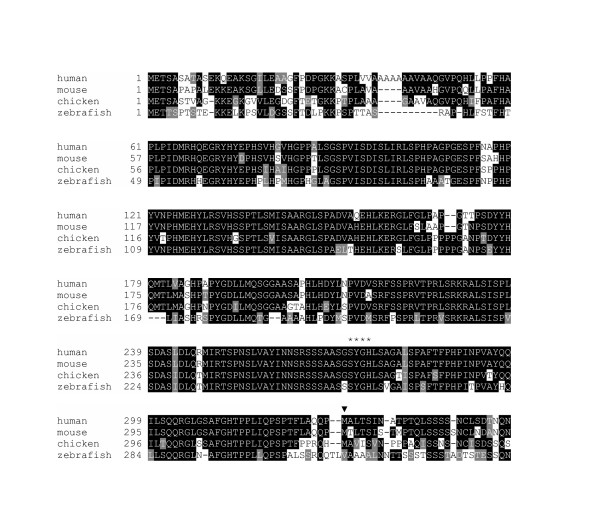
**Evolutionary conservation of GLI2**. ClustalW multiple alignment of Gli2 orthologs from human, mouse, chicken and zebrafish species (only N-terminal parts are shown). In the alignment, zebrafish yot protein (encoded by *you-too*) is shown. Su(fu) binding site SYGH is indicated by asterisks. The initiator methionine of human GLI2 defined by Tanimura et al. [32] is marked by arrowhead.

GLI2 protein is conserved throughout vertebrate evolution, showing high degree of sequence identity to its orthologs from mouse (83%)[34], chicken (68%)[35] and zebrafish (56%)[36] (IP unpublished data). Conservation of the N-terminus (residues 61–284), containing putative repressor domain [12], is even more striking – 94% identity with mouse, 87% identity with chicken, and 78% identity with zebrafish (Fig. 2).

### *GLI2 *has two alternative 5' noncoding exons and extension in the 3' UTR

**Figure 3:**
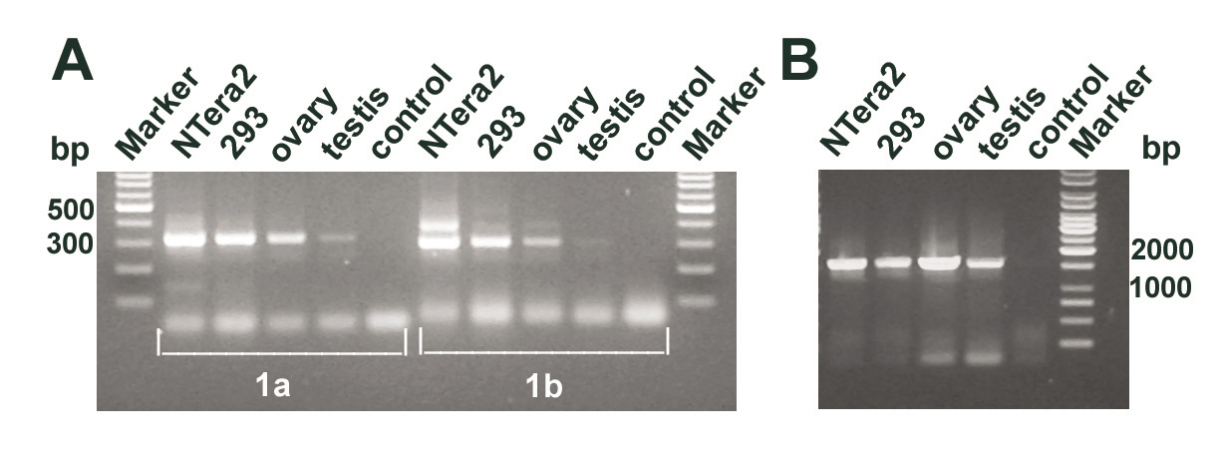
**Revised structure of the human *GLI2 *has two alternative 5' noncoding exons and extension in the 3' UTR**. (A) RT-PCR with primers derived from exons 1a-3 and exons 1b-3 are shown (adjoined frames 1a and 1b, respectively). Amplification products, 315 bp for exons 1a-3 and 295 bp for exons 1b-3 are shown. The origin of a 400 bp PCR product in 1b-3 reaction of NTera2D1 is unknown. (B) RT-PCR with primers annealing to the 3' UTR. A1616 bp amplification product is shown. Complementary DNAs used were derived from tissues and cell lines indicated on top. Control, no cDNA.

Comparison of human and mouse *Gli2 *transcripts (e.g. [GenBank: NM_030379 and CN536241] with the human genomic sequence predicted two alternative 5' noncoding exons in the human *GLI2 *locus, designated exon 1a and 1b (Fig. 1). Exon 1a is highly conserved between human, mouse (corresponds to exon 1 in mouse *Gli2 *in Fig. 1), rat and dog, showing 75–83% identity at the nucleotide level (data not shown). It is about 60 kb upstream of the exon 2 (the first coding exon). Exon 1b is located about 5 kb upstream of exon 2 and corresponds to the 5' sequence of the original *GLI2 *mRNA, cloned from HTLV-1-infected Hut102 cells [32]. To determine whether exons 1a and 1b are transcribed *in vivo*, we amplified the *GLI2 *5' region from human cell line and tissue cDNAs using forward and reverse primers annealing to exons 1a or 1b and 3, respectively. Amplification products of predicted size and sequence were obtained for exon 1a (315 bp) as well as exon 1b (295 bp) using cDNAs derived from ovary, testis, teratocarcinoma cell line NTera2D1 and human embryonic kidney cell line HEK293 (Fig. 3A). This result shows that both alternative first exons are used *in vivo*. Although we did not quantify the level of *GLI2 *mRNA in different human tissues, *GLI2 *transcripts containing exon 1a seemed to be more abundant than those containing exon 1b. In summary, these results show that human *GLI2 *locus harbors two alternative 5' noncoding exons, one of which is highly conserved and has not been described before. The *GLI2 *cDNA sequences encompassing exons 1a and 1b have been deposited in GenBank under accession numbers DQ004397 and DQ004398, respectively.

The published human *GLI2 *mRNAs [32] lack about two thirds of the 3' UTR (in exon 14) when compared to the mouse *Gli2 *3' UTR. Comparison with human ESTs [GenBank: AI822132, BX103004 and AI089685] and genomic sequence (NT_022135) predicts that the human *GLI2 *mRNA has a 1822 nt-long 3' UTR containing polyadenylation signal ATTAAA. This signal and its surrounding region are well conserved between human and mouse *Gli2 *suggesting their requirement for polyadenylation. This fact is also supported by two *GLI2 *ESTs that terminate with poly(A) sequences 16 nt downstream of the signal [GenBank: CA430900 and AI204540].

**Figure 4:**
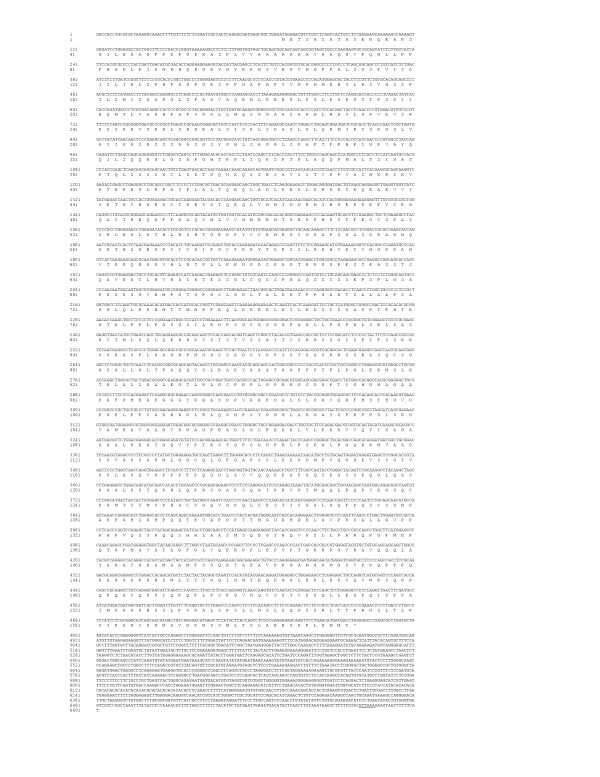
**Structure of the human *GLI2 *mRNA and its translation**. Human *GLI2 *mRNA containing conserved 5' noncoding sequence of 69 nt (exon 1a) and complete 3' UTR (1822 nt) are shown. Poly(A) signal is underlined.

To confirm the presence of the predicted 3' UTR in the *GLI2 *transcripts, RT-PCR with 3' UTR-specific primers was carried out. Amplification products of the predicted size (1616 bp) and sequence were obtained for cDNAs derived from gonadal tissues and two human cell lines (Fig. 3B). This result shows that human *GLI2 *mRNA has an extended 3' UTR as predicted from the bioinformatic analysis. Another potential polyadenylation signal AATAAA, located 1119 nt upstream, has been described earlier [32]. However, this may rather represent a cloning arfefact, since this motif is followed by an A-rich region (AAAAAGGAAAGAAAAAA) known to cause oligo-dT mispriming during cDNA synthesis [37] (Fig. 4). Futhermore, this A-rich sequence is not conserved in mouse *Gli2 *gene. The revised structure of the human *GLI2 *mRNA containing complete ORF and its translation is shown in Fig. 4.

### Identification of novel alternatively spliced forms of *GLI2 *mRNA

**Figure 5:**
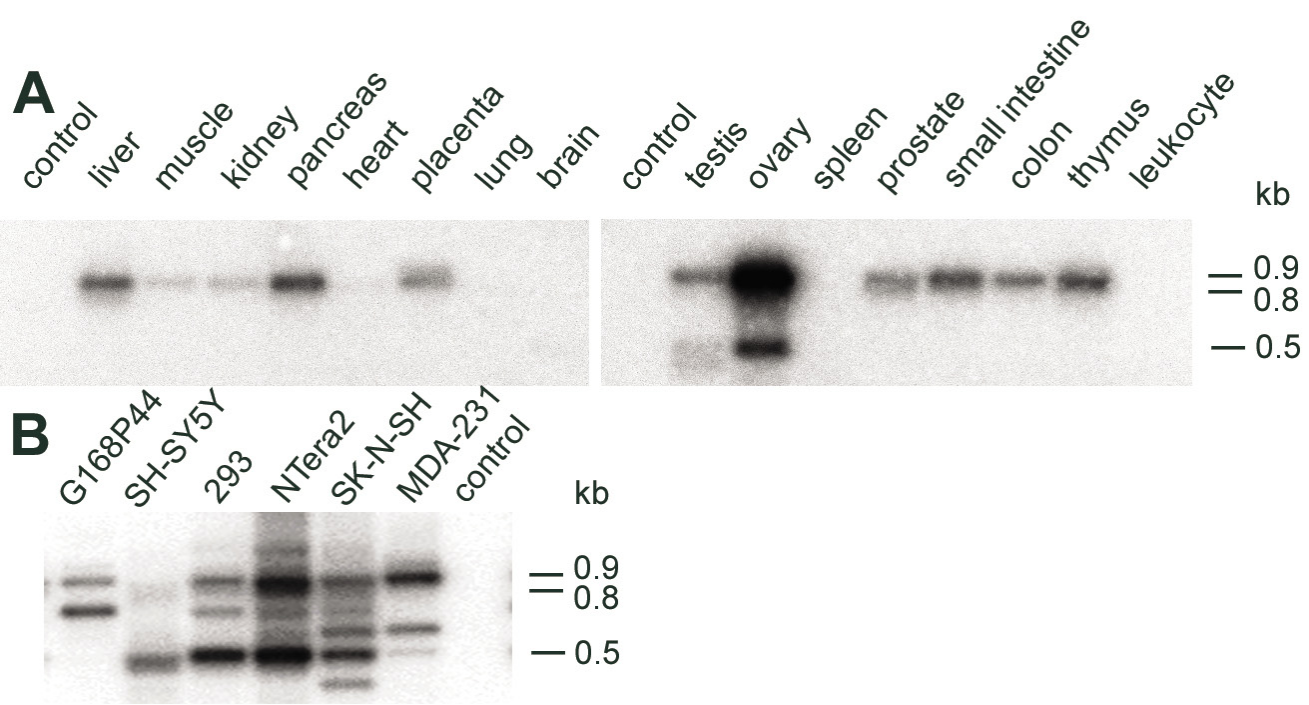
**Tissue-specificity of *GLI2 *mRNA and its alternatively spliced forms**. Southern blot analysis of the PCR products amplified from cDNAs derived from (**A**) normalized multiple tissue panels and (**B**) different cell lines (indicated on top of the panel) using *GLI2*-specific primers derived from exons 2 and 7. The products were hybridized with a 918 nt-riboprobe encompassing *GLI2 *exons 2–7. Bars on the right indicate sizes of PCR products corresponding to *GLI2 *mRNA and its alternatively spliced forms *GLI2*Δ3 and *GLI2*Δ4–5 (0.9, 0.8 and 0.5 kb, respectively). A shorter and longer exposure were used to determine the presence of minor transcripts corresponding to *GLI2*Δ3 (0.8 kb) in ovary and testis (panel A). Note the complex pattern of alternatively spliced products observed for cell lines (panel B) that is most likely due to promiscuous splicing (not analyzed in detail). Control, no cDNA.

We decided to study *GLI2 *expression in different human tissues because our gene/mRNA structure analysis predicted the existence of mRNA alternatively spliced forms. To determine the expression profile of human *GLI2 *mRNA, we carried out PCR with primers derived from exons 2 and 7. PCR products of the expected size (918 bp) were observed for a number of commercial cDNAs and different human cell lines. Figure 5A shows that *GLI2 *mRNA is strongly expressed in the ovary, testis, pancreas, liver, small intestine and thymus. While low level of expression was observed for a number of tissues (e.g., placenta, prostate and colon), almost no expression was detected in heart, brain and peripheral blood leukocytes. Three 0.9 kb products of identical size obtained from prostate, ovary and spleen cDNAs were selected for cloning and sequencing. Sequencing of two randomly selected clones from each cloning confirmed that human *GLI2 *mRNA contained exons 3–6, as predicted from the gene structure (Fig. 1). The cloned sequence encompassing exons 2–7 is available in GenBank under accession number AY493737.

**Figure 6:**
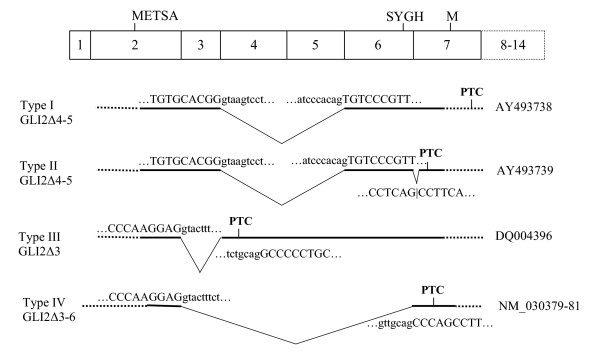
**Alternative splicing in the 5' end of *GLI2 *mRNA**. Exons corresponding to cloned and/or sequenced splice variants are marked by bold lines. Exon and intron splice junction sequences are designated by upper and lower case letters, respectively. Alternatively spliced forms I-III arising via exon skipping and different exon 6–7 junction usage (described in this study) are compared with the previously published sequence (Type IV) [32]. For each alternatively spliced form, GenBank accession number is shown on the right. Translation of these sequences initiated from ATG codon located in exon 2 (METSA) terminates with premature termination codons (PTC). Location of initiator codon described previously [32] is shown in exon 7 (M). Location of the SUFU binding site (SYGH) in exon 6 is shown.

We also detected three minor RT-PCR products corresponding to *GLI2 *transcript variants approximately 100 and 400 bp shorter than *GLI2 *mRNA described above. These transcripts were present exclusively in ovary, testis and different cell lines (Fig. 5). Sequencing of 2–3 individual cDNA clones (RT-PCR cloning from ovary, testis and NTera2D1 cells) corresponding to these variants showed that they represented three different types of alternative splicing (Fig. 6). Type I clones had exon 3 spliced to exon 6. Type II clones showed similar skipping of exons 4 and 5, but had a different splice acceptor site between exons 6 and 7. Type III clones had exon 2 spliced to exon 4. Representative sequences of each type of splicing are available in GenBank under accession numbers AY493738, AY493739 and DQ004396. These results show that in the human tissues, *GLI2 *mRNA may be represented by three different alternatively spliced forms. For all *GLI2 *mRNA alternatively spliced forms described, the major ORF starting from the exon 2 with the sequence METSA (Fig. 6) was followed by premature termination codon. Thus, translation from the alternatively spliced forms is possible only from downstream initiator codons in frame with the main ORF.

### Alternative splicing of *GLI2 *mRNA is responsible for the synthesis of GLI2 protein isoforms with different activities

**Figure 7:**
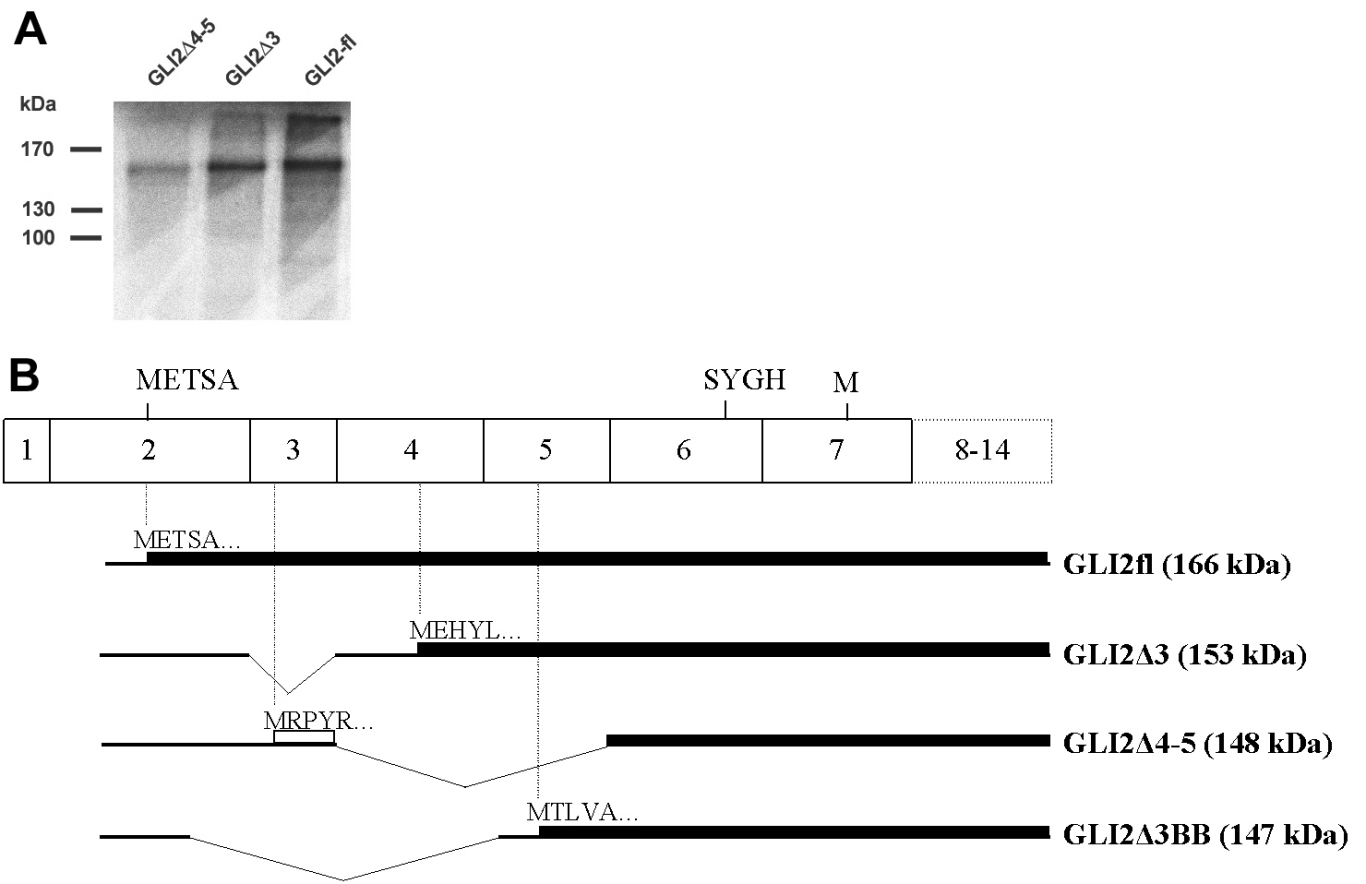
***In vitro *translation of the *GLI2 *mRNA and its alternatively spliced forms**. (**A**) SDS-gel analysis of [^35^S]-methionine labeled products translated with rabbit reticulocyte lysate. Constructs used are indicated on the top. Molecular weight marker positions are shown on the left. (**B**) Schematic representation of the translation products of the splice forms (shown on the right, with predicted molecular weight in parenthesis). mRNAs are shown by bold lines and their translation products are drawn by solid boxes. Open box represents translation in a different reading frame. The predicted N-terminal sequences (5 aa) are shown.

We hypothesized that splicing within the first seven exons may be involved in the exclusion or inclusion of the repressor domain of GLI2 protein. To test if alternatively spliced isoforms *GLI2*Δ3 and *GLI2*Δ4–5 can produce functional proteins, the corresponding cDNAs were subcloned into pCDNA3 expression vector and tested for the production of proteins using translation *in vitro*. Fig. 7A shows that *GLI2*Δ4–5 and *GLI2*Δ3 generate N-terminally truncated proteins with approximate sizes 155 kDa and 160 kDa, respectively. Because *GLI2 *ORF starting from the AUG located in exon 2 ended with premature termination codon, translation of the alternatively spliced forms of *GLI2 *was possible only from downstream AUG codons (Fig. 7B). The low protein yield obtained in the case of *GLI2*Δ4–5 was apparently due to inefficient translation initiation.

**Figure 8:**
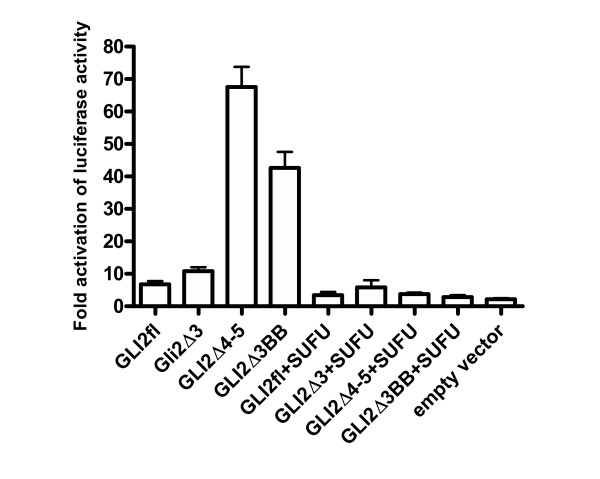
**Expression of the *GLI2 *and its alternatively spliced forms produces protein isoforms with different activities**. Transactivation assay with luciferase reporter plasmid (12XGLIluc), various *GLI2 *and SUFU constructs was carried out as described in Methods section using COS-7 cell line.

To determine the activation or repression effect of proteins produced from *GLI2 *mRNA and its alternatively spliced forms, each construct (*GLI2*fl, *GLI2*Δ3 and *GLI2*Δ4–5) was co-transfected with 12XGLIluc reporter plasmid into COS-7 cells. In this transactivation assay, *GLI2*Δ3 activator effect was comparable to that of the *GLI2*fl (Fig. 8). However, *GLI2*Δ4–5 showed about 10-fold increase in the reporter activity, suggesting that the enhanced activation was due to the loss of repressor activity, i.e. excision of repressor domain (or part of it) by alternative splicing.

Transactivation experiments repeated with PTCH promoter, containing two GLI2 binding sites [19], yielded similar results, although in this case about 3-fold enhancement was detected (data not shown). Transcriptional activity of all constructs was significantly suppressed by co-transfection with SUFU construct indicating that SUFU-binding domain, SYGH, encoded by exon 6 was intact in all constructs used. These data show that both *GLI2*Δ4–5 and *GLI2*Δ3 generate alternatively spliced forms that can be translated into active proteins. Difference in their activities is most likely caused by the N-terminal sequence MEHYLRSVHSSPTLSMISAARGLSPADVAQEHLKERGLFGLPAPGTTPSDYYHQMTLVAGHPAPYGDLLMQSGGAASAPHLHDYLNPVD, encoded by *GLI2*Δ3 and missing in *GLI2*Δ4–5. We suspected that this 89 aa region (or part of it, depending on translation initiation site used) might contain critical sequences required for GLI2 repressor activity. To map these sequences more precisely, we deleted the first 32 aa of the N-terminal sequence encoded of *GLI2*Δ3 by generating *GLI2*Δ3BB. Expression of this construct most likely produces an N-terminally truncated protein with translation initiation from the sequence MTLVAG located 54 aa downstream with respect to the *GLI2*Δ3 translation initiation. This prediction was also supported by *GLI2*Δ3BB translation *in vitro *(data not shown). In the transactivation assay, *GLI2*Δ3BB showed enhanced activation reaching about 70% level of that of *GLI2*Δ4–5 (Fig. 8), suggesting that 54 aa encoded by exons 4 and 5 were critical for the repressor activity. In summary, our results show that alternative splicing involved in the deletion of the repressor domain encoded by exons 4 and 5 is responsible for the enhanced activation of GLI2 protein.

## Discussion

In this study we describe the structure of the human *GLI2 *gene and its expression in different human tissues. A detailed comparison between human and mouse *Gli2 *structures allowed us to revise the structure of human *GLI2 *[32,33] by introducing a novel noncoding exon (1a) and extending the 3' UTR. Our data support the earlier finding of Roessler et al. [33] that human *GLI2 *contains exons 3–6 encoding repressor domain. This domain is highly conserved in evolution and has a major impact on the modulation of GLI2 transcriptional activity. As inferred from the studies on Ci and Gli3, the most likely mechanism that separates repressor and activator domains is proteolytic processing [13,15]. Here we show for the first time that in addition to proteolytic processing, alternative splicing may be another important regulatory mechanism which causes deletion of the major part of repressor domain and thus is responsible for the enhanced activation of human GLI2. In theory, translation from the downstream initiator codon observed in the alternatively spliced form *GLI2*Δ4–5 is rather inefficient. Nevertheless, the activation effect observed (about 10-fold) is significant, although less protein is produced from *GLI2*Δ4–5 compared to *GLI2*fl.

Previous studies of others [12] showed that the removal of the N-terminal region (residues 1–279 encoded by exons 2–6) resulted about 10-fold enhancement of the transcriptional activity of mouse Gli2. Similarly, expression of truncated human GLI2 lacking N-terminal 328 aa, encoded by exons 2–6, demonstrated substantial increase (about 10 to 30-fold) in transcriptional activity of GLI2 [33]. Also, cotransfection experiments showed that the repressor domain encoded by exons 2–6 is involved in the dominant-negative activity of disease-associated GLI2 mutants [33].

Taken together, all these results show that the N-terminal region of either mouse or human GLI2 contains a domain with transcriptional repressor activity. While all these results suggest that repressor domain of mouse and human GLI2 is encoded by exons 2–6, our results show the the removal of exons 4–5 alone can affect transcriptional activity of GLI2. Additional mapping of the repressor activity showed that a critical 54 aa-long sequence is encoded by exons 4 and 5. Therefore, our results strongly suggest that alternative splicing may be involved in the regulation of the synthesis of GLI2 proteins with or without repressor activities. Interestingly, we have detected several *Gli2 *mRNA splice forms, including *Gli2*Δ4–5 and *Gli2*Δ3 in mouse embryos, indicating that alternative splicing of *Gli2 *pre-mRNA may be evolutionarily conserved (Hanna Tulmin, Pille Pata, PK and IP, unpublished data).

Previous studies [6,32] have suggested that human *GLI2 *mRNA may exist in at least four different isoforms, which can be detected in tumor cell lines or tissues. Here we have analyzed alternative splicing in the 5' region of *GLI2*, encompassing exons 1–7, in normal human tissues. We found that exons 3–5 are involved in the alternative splicing and corresponding alternatively spliced forms (skipping exons 3 and 4–5) were exclusively found in adult ovarian and testicular tissues, raising a question about potential role of GLI2 acting solely as an activator of germ cell development. It should be noted, that knock-in mice expressing full-length *Gli2 *cDNA from the endogenous *Gli2 *locus are normal and viable, arguing against the role of alternative transcripts in normal mouse development [25]. Our attempts to detect alternatively spliced forms of *GLI2 *mRNA, in which exons 2 and 7 were spliced together (*GLI2*Δ3–6), as described earlier [32], have not been successful. It is likely that different alternatively spliced forms are expressed in tumor cell lines or tissues. However their origin remains unclear. It is also important to note that a highly conserved motif SYGH involved in the interaction of GLI2 with SUFU [21] is encoded by exon 6. Because this exon is lost in the spliced form *GLI2*Δ3–6, it is possible that its expression gives rise to a GLI2 protein escaping repression and/or sequestration effects of SUFU. We believe that the loss of SUFU binding site of GLI2 protein may have important implications in the regulation of Shh signaling pathway. However, to prove this possibility, additional experiments are required.

We have shown that human *GLI2 *contains two alternative noncoding 5' exons 1a and 1b. This feature typically suggests the usage of alternative promoters and thus adds another layer of complexity to the regulation of human *GLI2*. It remains to be explored how these promoters can influence the biological function of human *GLI2*.

## Conclusion

We report here the revised structure of human *GLI2 *gene. We present evidence that alternative splicing regulates the transcriptional activity of *GLI2*. Our data suggest that in addition to proteolytic processing, alternative splicing may be another important regulatory mechanism for the modulation of repressor and activator properties of GLI2 protein.

## Methods

### Biocomputational analysis

Comparison of the mouse and human *GLI2 *genomic DNAs and mRNAs was carried out by SPIDEY [38]. Repetitive DNA elements were identified by RepeatMasker (A.F.A. Smit and P. Green, unpublished data). Previously published *Gli2 *mRNA sequences were extended at their 5' and/or 3' termini using overlapping expressed sequence tags (ESTs) derived from the searches of GenBank database using MEGABLAST [39]. The extended mRNA structures were mapped to the genomic structure by SPIDEY. All translations and mRNA/cDNA sequence comparisons were done with DNAMAN Version 4.0 (Lynnon BioSoft). Mouse, chicken and zebrafish Gli2 protein sequences were derived from databases [GenBank: XP_136212, XP_422086, and AAD18135].

### Accession numbers

Sequence data described in this study were deposited into GenBank under accession numbers AY493737, AY493738, AY493739, DQ004396, DQ004397, and DQ004398.

### Reverse transcription, DNA amplification and cloning

PCR of the normalized multiple tissue cDNA (MTC) panels I-II (BD Biosciences) and cDNAs prepared from different human cell lines (oligo dT and random priming) was carried out with primers designed into exons 2 (GCCTCCGAGAAGCAAGAAGC) and exon 7 (TGGTGTGTGTCCAAAGGCTGA) using the following temperature profile: 95°C 30 s, 55°C 30 s and 72°C 1 min for 35 cycles. The following human cell lines were used: neuroblastomas SH-SY5Y (ATCC Number: CRL-2266) and SK-N-SH (ATCC Number: HTB-11); mammary gland adenocarcinoma MDA-MB-231 (ATCC Number: HTB-26); glioma G168P44 (a gift from Andres Veske); teratocarcinoma NTera2D1 (ATCC Number: CRL-1973); embryonic kidney HEK293 (ATCC Number: CRL-1573). All PCR products encompassing *GLI2 *exons 2–7 or their alternatively spliced forms were analyzed by agarose gel electrophoresis, transferred to Hybond N^+ ^membrane (Amersham Biosciences) and hybridized with a 918 nt ^32^P-labeled riboprobe prepared from the cloned *GLI2 *cDNA containing exons 2–7 [40]. After gel-elution, PCR products were cloned into *Sma*I site of the pBluescript SK+ vector (Stratagene) by blunt-end ligation. Recombinant DNAs were isolated and both strands of inserts were sequenced using T3 and T7 primers.

Cloning of the human *GLI2 *cDNA containing complete ORF was carried out as follows. First strand cDNA was synthesized with SuperScript III Reverse Transcriptase (Invitrogen) using total RNA isolated from human teratocarcinoma cell line NTera2D1 (ATCC Number: CRL-1973). PCR amplification of cDNA was carried out in two separate experiments. A 2481 bp 5' terminal fragment was generated with primers D3 (TGCTGCTTTACCGACACATC) and R6A (GGAGGAGCGGCGGCTCACG), and a 2290 bp 3' terminal fragment was generated with primers D6 (GGCATCTCCCCCTACTTCTC) and Rev3 (TCTAGGTCATCATGTTCAGGA). PCR conditions were the same as in MTC panel amplification reaction (see above), except that annealing temperature was 55°C and extension time 4 min. Both fragments obtained were cloned into *Sma*I site of the pBluescript SK+ vector. To facilitate blunt-end cloning into expression vector, cloned 5' and 3' terminal fragments together with regions derived from multiple cloning site of the vector were amplified (10 PCR cycles) with a combination of T7 promoter primer and R6A, and T3 promoter primer and D6, respectively. Both gene-specific primers were phosphorylated at 5' termini. The obtained 5' and 3' fragments were joined by blunt-end ligation, digested with *Hin*dIII and *Xba*I and cloned into *Hin*dIII-*Xba*I linearized pCDNA3 expression vector. The final construct, *GLI2*fl, contained complete *GLI2 *ORF and its structure was verified by sequencing. Constructs *GLI2*Δ3 and *GLI2*Δ4–5 were generated by replacing a region flanked by *Bam*HI and *Bgl*II sites (encompassing exons 2–9) in *GLI2*fl with fragments obtained from alternatively spliced forms (Δ3 and Δ4–5) cloned in pBluescript SK+ vector. Cloning of alternatively spliced forms by RT-PCR was analogous to that described above, except that the following primers derived from exons 2 and 9 were used: GCCTCCGAGAAGCAAGAAGC and ACCTCAGCCTCCTGCTTACA. Construct *GLI2*Δ3BB was generated from *GLI2*Δ3 by deleting a 257 bp fragment with restriction enzymes *Bam*HI and *Bsp*T1 and blunting the ends with Klenow polymerase. All constructs were verified by DNA sequencing.

To analyze expression of alternative first exons 1a and 1b in human tissues and cell lines, PCR was carried out with the forward primers annealing to exon 1a (GGCCACCTGCGTGCTAGAG) or 1b (CCGACACATCAAAGAGCAAGGATTG) and the reverse primer annealing to exon 3 (ACCGTGGACAGAATGAGGCT). We used the first-strand cDNAs from human ovary and testis (BD Biosciences), and cDNAs derived from NTera2D1 and HEK293 cell lines. These cDNAs were synthesized with Superscript III using total RNA. Amplification was conducted at 95°C 30 s, 55°C 30 s and 72°C 1 min for 40 cycles.

To confirm the presence of the predicted 3' UTR in *GLI2 *transcripts, RT-PCR with primers TTTATGGGCATCCTCTCTGGT and GCATGTCATCTCAATTCATAGCA was used for the amplification of a 1616 bp fragment derived from a region located 27 bp upstream to the polyadenylation signal (ATTAAA). The amplification profile used was identical to that described for exons 1a/1b-3 (above), except that the extension step was 2 min. To exclude the amplification from genomic DNA, RT minus reaction was used as a negative control.

### *In vitro *translation and transactivation assay

In vitro translation assays were carried out using TNT^® ^Quick Coupled Transcription/Translation Systems (Promega). COS-7 cell line was used for the luciferase reporter assay. Transfection of cells plated on 24-well plates reaching cell density about 80% was carried out with 0.5 μg of *GLI2 *construct (*GLI2fl*, *GLI2*Δ3, *GLI2*Δ3BB and *GLI2*Δ4–5) 0.3 μg of SUFU DNA or empty vector, 0.1 μg 12XGLIluc reporter plasmid and 0.1 μg of pCMV-β-gal using FuGene (Roche) according to the manufacturer's instructions at DNA to FuGene ratio of 1:3 (w/v). SUFU and reporter plasmid used in this study have been described previously [19]. pCDNA3 plasmid DNA was added to the transfections as needed to achieve the total amount of plasmid DNA per transfection. After 24 h the medium was replaced with low serum media (0.5% calf serum) and cells were incubated for an additional 24 h. Subsequently cells were lysed and luciferase activity was measured with a luciferase kit from Tropix (Bedford) according to the manufacturer's instructions using an Ascent Fluoroscan combined fluori- and luminometer (Thermo Lab-Systems). Luciferase activities were normalized with respect to parallel β-gal activities, to correct for differences in transfection efficiency. β-gal assays were performed using Galacto-Light/Galacto-Light Plus Systems (Tropix) according to the manufacturer's instructions. All experiments were repeated at least three times.

## Authors' contributions

MS carried out bioinformatic studies, RT-PCR experiments, synthesis of the *GLI2 *cDNA, *in vitro *translation and drafted the manuscript. ON carried out transfection experiments and participated in the design of experiments. IP did sequence alignment of Gli2 proteins and RT-PCRs of *GLI2 *exons 1a/1b-3 and 3'UTR. EV participated in the design of the study and helped to draft the manuscript. PK participated in the design and coordination of the study. All authors read and approved the final manuscript.
